# Video-based Goniometer Applications for Measuring Knee Joint Angles during Walking in Neurological Patients: A Validity, Reliability and Usability Study

**DOI:** 10.3390/s23042232

**Published:** 2023-02-16

**Authors:** Monica Parati, Matteo Gallotta, Beatrice De Maria, Annalisa Pirola, Matteo Morini, Luca Longoni, Emilia Ambrosini, Giorgio Ferriero, Simona Ferrante

**Affiliations:** 1 Department of Electronics, Information and Bioengineering, Politecnico di Milano, 20133 Milan, Milan, Italy; 2 Istituti Clinici Scientifici Maugeri IRCCS, 20138 Milan, Milan, Italy; 3 Istituti Clinici Scientifici Maugeri, 20851 Lissone, Monza Brianza, Italy; 4 Istituti Clinici Scientifici Maugeri IRCCS Tradate, 21049 Tradate, Varese, Italy; 5 Department of Biotechnology and Life Sciences, University of Insubria, 21110 Varese, Varese, Italy

**Keywords:** smartphone, goniometer, knee range of motion, measurement properties, usability, stroke, Parkinson’s disease, rehabilitation

## Abstract

Easy-to-use evaluation of Range Of Motion (ROM) during walking is necessary to make decisions during neurological rehabilitation programs and during follow-up visits in clinical and remote settings. This study discussed goniometer applications (DrGoniometer and Angles - Video Goniometer) that measure knee joint ROM during walking through smartphone cameras. The primary aim of the study is to test the inter-rater and intra-rater reliability of the collected measurements as well as their concurrent validity with an electro-goniometer. The secondary aim is to evaluate the usability of the two mobile applications. A total of 22 patients with Parkinson’s disease (18 males, age 72 (8) years), 22 post-stroke patients (17 males, age 61 (13) years), and as many healthy volunteers (8 males, age 45 (5) years) underwent knee joint ROM evaluations during walking. Clinicians and inexperienced examiners used the two mobile applications to calculate the ROM, and then rated their perceived usability through the System Usability Scale (SUS). Intraclass correlation coefficients (ICC) and correlation coefficients (corr) were calculated. Both applications showed good reliability (ICC > 0.69) and validity (corr > 0.61), and acceptable usability (SUS > 68). Smartphone-based video goniometers could be used to assess the knee ROM during walking in neurological patients, because of their acceptable degree of reliability, validity and usability.

## 1. Introduction

Accurate and precise measurement of knee Range Of Motion (ROM) during a functional movement such as walking is essential for many aspects of rehabilitative decision making, including the identification of joint limitations, the assessment of treatment response, and the use for research purposes [1,2,3]. For the patients themselves, it also allows them to appreciate their progress, and could be adopted as a goal for rehabilitation [4]. To date, marker-based three-dimensional (3D) motor caption systems are still the gold standard to provide the kinematic and kinetic parameters of human gait, including knee joint angles during flexion/extension movements [5,6,7,8]. Passive reflective markers are placed on the subject’s landmarks using standardized gait analysis protocols [9]. Multiple cameras and sophisticated software calculate the trajectories of these markers and produce quantified, valid, and reliable results over a short-distance walking test [9]. However, these systems require a stationary laboratory, expensive resources, experienced personnel, and substantial patient commitment. An alternative cheaper solution to quantify joint angles is represented by electro-goniometers [6]. They consist of two end blocks connected by a protective string, housing a composite wire with a series of strain gauges mounted around the wire circumference [6]. For measuring knee joint angles during flexion and extension movements, the electro-goniometer is attached by an operator on the lateral part of the knee, with its center settled on the femoral condyle and their end blocks parallel to the lateral malleolus, and the long axis of the femur and the greater trochanter [8]. From the electro-goniometer signals, joint angles are easy to derive without sophisticated software [6]. However, their correct placement might be difficult for some joints such as the hip. 

Wearable systems, based on Inertial Measurement Units (IMUs) [10,11], and video-based marker-less technologies, which exploit depth cameras [12,13], are becoming emerging and viable options for measuring joint kinematics. These emerging systems are relatively inexpensive, need lower preparation time than gold-standard marker-based motion capture systems, and have great potential for assessment outside of a clinical or laboratory setting. However, they require ad hoc software and dedicated hardware to be worn or installed in a defined environment. These factors might impact their large-scale use and limit their feasibility for long-term monitoring.

In recent years, an increasing number of mobile applications have been tested to provide instantaneous, easy-to-use readings of the joint ROM, and some are currently available in mobile application stores [14,15]. 

Such applications adopt different functioning to achieve the same goal. Some apps require that the smartphone is fixed over the subjects’ skin to collect measures of joint angles using the built-in accelerometer, gyroscope, and magnetometer signals [14,16,17]. Other apps do not require that the smartphone is in direct contact with the subject’s skin because joint angles are drawn on digital photography [14,18,19]. Specifically, the latter app type offers additional benefits to accelerometer-based goniometric apps. Indeed, through their easy image storage and automatic transfer of angle data, they allow (i) multiple observers to measure an identical motion, (ii) observers to measure the same motion at different times, and (iii) observers to assess individuals located in a different place. This ability has made their use a more attractive option for remote monitoring and telerehabilitation [20]. Moreover, the widespread adoption of smartphones might favor their large-scale use and their feasibility for long-term applications.

There are several reports on the assessment of measurement properties of joint angle measurements collected by mobile applications. Two recent reviews by Keogh et al. [15] and Longoni et al. [14] identified more than thirty studies involved in the validation process of mobile apps for measuring joint angles. These reviews reported that most of the studies tested the measurement properties of joint angle measurements by accelerometer-based mobile apps. Moreover, these studies mainly collected validation data on healthy volunteers in static conditions. The review by Keogh et al. [15] specifically reported that all the studies using accelerometer-based goniometer apps tested the measurement properties of knee joint angles in the static condition, in the supine position or during a standing lunge. Koegh and colleagues also reported that validation testing of knee joints on pathological subjects was reported in less than 25% of the studies, and validation data on neurological patients were not currently available for knee angle measurements. To the best of our knowledge, these statements are also valid for image/video-based goniometer apps. The evaluation of the measurement properties of ROM measures using image/video-based mobile apps on pathological subjects during dynamic conditions is indeed limited, if not absent. Only one study tested the measurement properties of knee joint angles collected by a video-based goniometer app in dynamic conditions on healthy subjects [21].

Therefore, the current study aims to offer five novel contributions to the literature: (i) to examine the measurement properties of knee ROM measurements collected by two video-based goniometer apps in a dynamic conditions, i.e., during walking; (ii) to extend their validation on pathological subjects; (iii) to assess the intra-rater and inter-rater reliability of the two video-based goniometer apps; (iv) to assess the concurrent validity of the video-based goniometer apps with an electro-goniometer, a relatively inexpensive system used in clinical settings; and (v) to test the usability of mobile apps from clinical and non-clinical perspectives.

We hypothesized that there would be good agreement between measures collected by video-based goniometric apps and the electro-goniometer, and that the inter-rater and intra-rater reliability of the two goniometric apps would be good. Finally, we hypothesized that the mobile apps had acceptable usability for the ROM assessments. 

## 2. Materials and Methods

### 2.1. Study Design

This cross-sectional study was performed at the IRCCS Istituti Clinici Scientifici Maugeri (Milan and Lissone, Italy). Procedures were conducted according to the Declaration of Helsinki and approved by the Istituti Clinici Scientifici Maugeri ethics committee (2456CE, July 2020). Informed consent was obtained from all participants included in this study.

### 2.2. Participants

A convenience sample of patients with Parkinson’s disease (PD) and hemiparetic stroke (SK) undergoing neurological rehabilitation at IRCCS Istituti Clinici Scientifici Maugeri (Milan and Lissone, Italy) and a control group of healthy volunteers (HC) were recruited in this study. 

The inclusion criteria were participants (i) older than 40 years, and (ii) able to walk without physical assistance. The exclusion criteria were participants with (iii) assistive walking devices interfering with the video recording of the ankle, knee, and hip, and (iv) any other diseases interfering with gait and posture. 

Sociodemographic and clinical data were collected for every participant to examine their eligibility for the study and characterize the participants’ sample. The modified Rankin scale (mRS) was specifically collected for SK patients to describe their global disability during daily life activities [22]. It consists of a 6-point scale with possible scores ranging from 0 (no symptoms) to 5 (severe disability) [22].

For PD patients, the modified Hoenn and Yahr scale (mH&Y) was collected to describe the severity of the Parkinson’s disease symptoms [23]. This 7-point scale could range from 1 (symptoms with unilateral involvement only) to 5 (wheelchair-bound or bedridden unless aided) [23].

### 2.3. Examiners

Two physiotherapists (with more than 5 years of experience in neurological rehabilitation) and two inexperienced individuals in the field of physical and rehabilitation medicine were recruited to examine video recordings and perform knee joint ROM measurements on the two mobile apps. All the examiners were young adults (range: 22–41 years old), routinely used to electronic devices, and felt very confident in the mobile application usage.

### 2.4. Instrumentation

The Angles - Video Goniometer app (by Angles app developer Nathaniel Cochran) and the DrGoniometer app (by DrG app developer CDM, S.r.l.) are iPad/iPhone goniometric tools that use a virtual goniometer positioned on the smartphone screen to provide joint angle measurements on a photograph/video taken from the iPad/iPhone camera [18,21,24]. To the best of our knowledge, these two apps are the only ones that allow evaluations in dynamic conditions from video recordings and that showed a preliminary good validation in other contexts, namely in static conditions and/or in dynamic conditions on healthy populations [18,21,24].

**Figure 1:**
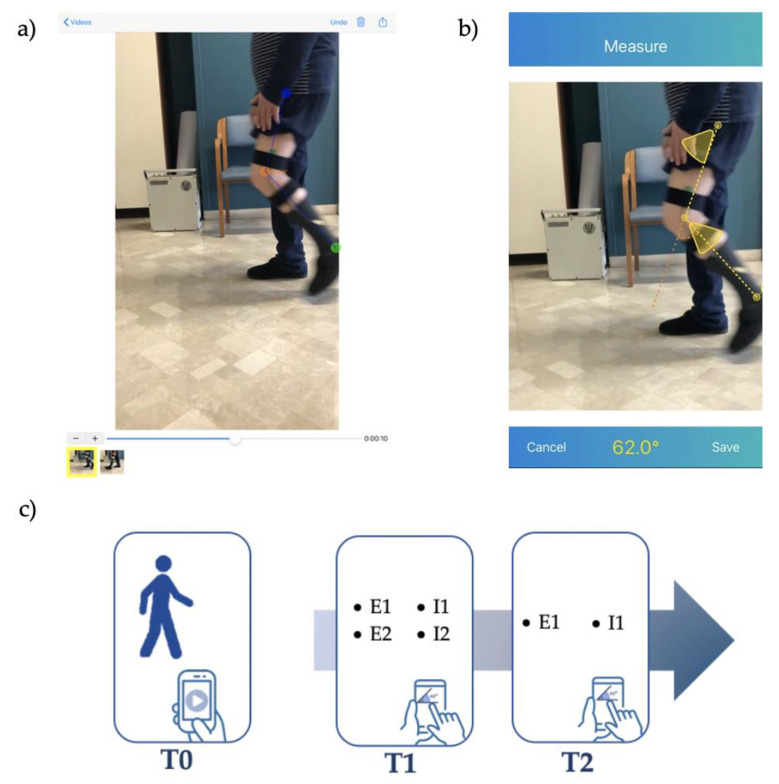
Screenshot images of the current version of Angles (**a**) and the DrGoniometer apps (**b**), and a schematic representation of the experimental study design composed of a testing patient’s session (T0) and two assessment sessions (T1, T2). The first assessment session (T1) was completed by two experienced examiners (E1, E2) and two inexperienced examiners (I1, I2). The assessments were repeated at T2 by one experienced examiner (E1) and one inexperienced examiner (I1) (**c**).

To perform a joint angle measurement using the Angles app, an examiner has to (i) acquire a video from the smartphone camera or import it from the photo library, (ii) scroll it across the screen using the horizontal scroll bar to select the video frame to evaluate, (iii) draw an angle placing three markers on the image (as shown in Figure 1a), and (iv) directly read the angle measurements on the screen and/or save these data on an office spreadsheet. The Angles app allows raters to select and analyze multiple video frames by importing a unique video (as shown in Figure 1a). In addition, the Angles app allows examiners to scroll the video and re-watch all the measured angles inside it. Once the examination was complete, the video with the measured angles was saved in an alphabetically ordered list. 

The DrGoniometer app has some different functionalities compared to the Angles app. Firstly, the main intended users of the app are clinicians. Indeed, its layout is structured to label and organize videos according to the patients’ names, joints, sides of the body, and clinical tests. These elements could notably be helpful to manage patients’ evaluations for clinicians, whereas the Angles app does not present these characteristics, but saves data in a simple alphabetic ordered list. The assessment process of DrGoniometer was relatively similar to that of the Angles app. The examiner could (i) acquire a video from the smartphone camera or import it from the photo library, and (ii) select the video frame to evaluate using a horizontal scroll bar. A virtual goniometer with three cursors then appears on the screen and the examiner could move it to define the joint angle. Once the examination is complete, the DrGoniometer app does not store the entire video, but only saves an image with the computed angles. Therefore, the DrGoniometer app does not allow examiners to simultaneously analyze multiple angles inside a single video.

Both apps allow users to display or hide the angle measures from the app settings. Both apps computed joint angles using the Pythagorean theorem and the law of cosines [21]. 

An electro-goniometer (Biometrics Ltd., Newport, UK) was used to establish the concurrent validity of the joint angles with the two mobile applications. 

### 2.5. Experimental Procedure

Following the recruitment process, the subjects were asked to stand in their usual posture, while a physiotherapist applied and fixed the electro-goniometer on the lateral part of the knee across the approximate knee joint center of rotation using double-sided tape and adjustable Velcro straps. Then, the participants were asked to walk at their self-selected comfortable speed and complete at least three gait cycles. A gait cycle is defined as the time between two consecutive heel strike events of the same foot. 

A smartphone (iPhone 8, Apple Inc., Cupertino, CA, USA) was vertically placed on a level surface lateral to the participants’ walking direction and perpendicular to the ground. Once the participants started walking, the smartphone recorded the participants in the sagittal plane using the smartphone camera. 

### 2.6. Data Analysis

The outcome measure of the study was the ROM of the knee joint during walking, assessed as the difference between the maximum flexion and maximum extension angles during a gait cycle. The analyses were performed on the paretic leg for SK patients and the right dominant leg for HC and PD patients. The right leg was arbitrarily chosen because the examined participants did not present evident laterality of the motor symptoms.

To compute ROM measurements using the apps, all the examiners followed a standardized procedure: each one watched the recorded videos on a tablet (iPad Pro 11′ v2, Apple Inc., Cupertino, CA, USA, operating system version: iPadOS 15). The examiners evaluated the same videos for both apps using a tablet pen (Apple Pencil 2, Apple Inc., Cupertino, CA, USA).

For each video to be analysed, the examiners were asked to select two video frames corresponding to the maximum knee flexion angle and the maximum knee extension angle, respectively, during the first completely visible gait cycle in the video recording. 

For each angle to be measured, examiners positioned the virtual goniometer on the tablet screen, placing the three cursors as follows: the central cursor on the retro-patella, the distal cursor on the middle of the lateral malleolus, and the proximal cursor on the hip along the estimated line of the femur, parallel with skin line [18]. 

One randomly selected experienced examiner (E1) and one randomly selected inexperienced examiner (I1) performed this procedure twice to evaluate the intra-reliability of collected measures. The time interval between the two repeated assessments (T1 and T2) was from one week to one month to limit biases due to recall issues between T1 and T2. A schematic representation of the study design is displayed in Figure 1c.

Moreover, all examiners (E1, E2, I1, I2) were blinded to other examiners’ assessments to possibly minimize biases in the score assignments. All examiners underwent a process of familiarization with the apps before starting the data analysis process. 

Joint angle measurements using the video goniometric apps were validated against a standard electro-goniometer whose good measurement properties have been previously established [6]. For the intra-rater reliability and concurrent validity, one rater for each examiners’ group (E1 and I1) was considered for the analysis.

At the end of the data analysis process, all the examiners completed the assessment of the perceived usability of the mobile apps through the system usability scale (SUS), which included 10 questions scored on a 5-point Likert-type scale from 1 (strongly disagree) to 5 (strongly agree) [25]. The SUS questionnaire is arranged to alternate between positive and negative statements to avoid habitual bias from the respondents. The score contribution for the odd items (the positive statements) is the item rating minus 1, and the contribution for the even items (the negative statements) is 5 minus the item rating. The total SUS score is calculated as the sum of all item scores multiplied by 2.5. Overall, the total SUS score could range from 0 to 100 [25]. A SUS score higher than 68 points indicates an acceptable level of system usability [26].

### 2.7. Statistical Analysis

All data were analyzed using SPSS (version 27, SPSS Inc., Chicago, IL, USA). Two-tailed significance level was set at alpha = 0.05 for all statistical analyses. A descriptive statistical analysis was conducted based on the mean values and standard deviations. A Shapiro–Wilk test was used to check for the normality of data. 

For intra-rater reliability analysis, a Student’s paired t-test or Wilcoxon signed-rank test was used, as appropriate, to verify the absence of systematic errors between the two repeated measures [27]. Intra-rater relative reliability was estimated by the intraclass correlation coefficient (ICC), using a two-way, mixed-effects, absolute agreement single-measurement model. To allow the error to be expressed in the original units of measurement, absolute reliability indices were computed. The standard error of measurement (SEM) was calculated using the ICC and the standard deviation of the two repeated measures (SEM = SD × $\sqrt{1-ICC}$). One SEM shows that the clinician may be 68% certain that the true measurement value lies within ±1 SEM of measurement from the clinical measurement, and 2 SEMs provide the clinician with a value with 95% confidence. Moreover, the minimum detectable change at the 95% confidence level (MDC) was computed as $\sqrt{2}$ × 1.96 × SEM, which represents the magnitude of change necessary to provide confidence that a change is not the result of random variation or measurement error. 

For the inter-rater reliability analysis, a Student’s paired t-test or Wilcoxon signed rank test, as appropriate, was applied to verify the absence of systematic errors between the raters [27]. Inter-rater relative reliability was estimated by two-way, random-effects, absolute agreement, single-measurement ICC model [28]. As occurs for intra-rater reliability, inter-rater SEM and MDC were computed. Because systematic differences are considered to be part of the measurement error, an ICC model with absolute agreement was preferred against an ICC consistency model for both reliability analyses [29].

For concurrent validity analysis, Pearson’s or Spearman’s rank correlation coefficients were calculated to determine the strength of the correlations regarding the knee joint ROM measured using the mobile apps and the comparison system. Bland–Altman analysis was carried out in order to extract the systematic mean bias and the 95% limits of agreement between the two systems [30]. The 95% limits of agreement were also calculated as mean difference ±1.96 × SD. The Bland–Altman plot displays a scatter plot of the average of the scores collected by the two analyzed systems versus their differences. If the agreement was good, then the differences should be randomly scattered around the zero difference reference horizontal line [30].

To interpret the study findings, ICC values were commonly interpreted as poor for values below 0.50, moderate between 0.50 and 0.75, good between 0.75 and 0.90, and excellent above 0.90 [28]. A SEM higher than 5° is considered large enough to mislead clinical interpretation [31]. For MDC, the threshold was 14°, obtained by a SEM threshold of 5° multiplied by $\sqrt{2}$ × 1.96 [31]. Correlation coefficients were interpreted considering the following classification: 0.90 to 1.00 = excellent, 0.70 to 0.89 = good, 0.50 to 0.69 = moderate, 0.30 to 0.49 = low, <0.30 negligible [32]. 

With a significance of 0.05 (alpha) and power of 0.20 (beta), and assuming a moderate correlation between measurements, a sample size of 20 joint measures for each group is required [33,34]. The minimum sample size was increased to 22, considering a possible drop-out rate of 10%.

## 3. Results

A total of 22 patients with Parkinson’s disease (18 males, mean age 72 (8) years), 22 post-stroke patients (17 males, mean age 61 (13) years), and 22 healthy subjects (9 males, mean age 45 (5) years) were included in the study. The sociodemographic and clinical characteristics of the participants are presented in Table 1.

Most of the enrolled SK patients were in the chronic phase (55%) of the disease (>6 months from stroke event) and had had an ischemic stroke (82%). The mean (standard deviation) score of the mRS scale was 2 (1), indicating that patients had a slight disability while performing activities of daily living.

The enrolled PD patients had a mean time since diagnosis equal to 5 (4) years, and had a mean (standard deviation) mH&Y score of 2 (1), revealing that Parkinson’s disease symptoms are present in both sides of the body, but did not involve balance deficits.

### 3.1. Intra-Rater Reliability

Table 2 includes the intra-rater reliability findings of the knee joint ROM collected by an experienced rater (E1) and inexperienced rater (I1) and their related mean ROM scores.

Overall, intra-rater reliability was found to be from good to excellent for ROM assessments (ICC > 0.79). No systematic biases between repeated assessments were present in the three examined populations for the Angles app and the DrGoniometer app (*p*-value > 0.05).

Acceptable absolute reliability was found in most of the ROM assessments (an SEM range of 2.0–3.4° for the experienced rater and 2.5–4.1° for the inexperienced rater, and an MDC range of 5.6–9.3° for the experienced rater and 9.0–11.4° for the inexperienced rater).

### 3.2. Inter-Rater Reliability

Table 3 displays the knee joint ROM angles collected by the two experienced examiners and two inexperienced examiners using the two mobile apps and the results of inter-rater reliability analysis.

There is some evidence of systematic differences between observers in the inexperienced examiners using the DrGoniometer app, specifically while assessing PD patients and HC. The ICC values were higher than 0.69 for all the assessment groups, suggesting that examiners had moderate to excellent inter-rater reliability in measuring the knee joint ROM, irrespective of the user experience and the goniometric device adopted.

When comparing the absolute reliability results among the examiners, the magnitude of error was largely similar for both the experienced and inexperienced groups in assessing knee joint ROM. The SEM ranged from 3.4° to 5.0°, while MDC ranged from 9.3° to 13.7°.

### 3.3. Concurrent Validity

**Figure 2:**
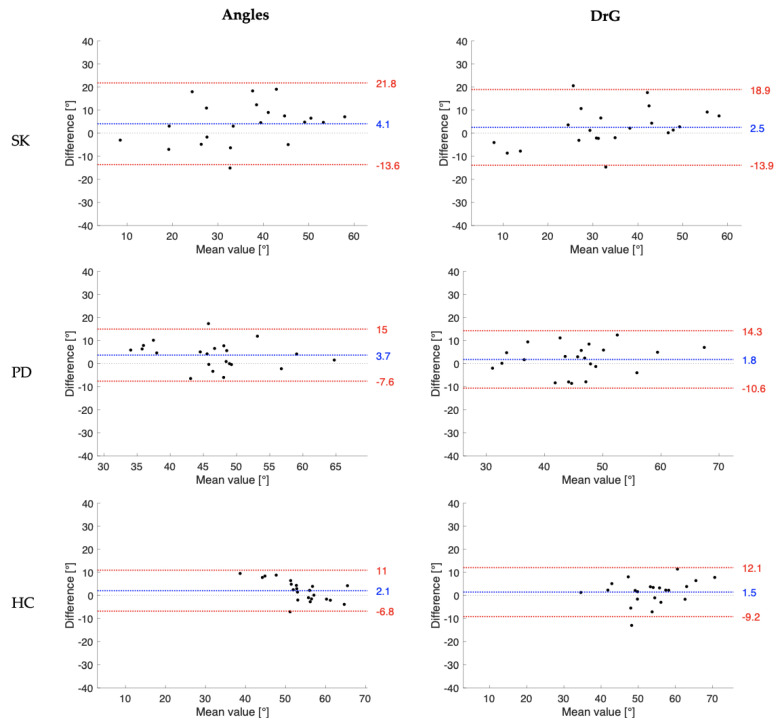
Bland-Altman plot of the knee range of motion of the three participant populations (SK: post-stroke patients, PD: patients with Parkinson’s disease, HC: healthy controls) collected by the experienced examiner (E1) using the Angles and the DrGoniometer applications. The blue line indicates the mean differences between the mobile app and electro-goniometer measures, and the red lines are the 95% lower and upper limits of agreement.

**Figure 3:**
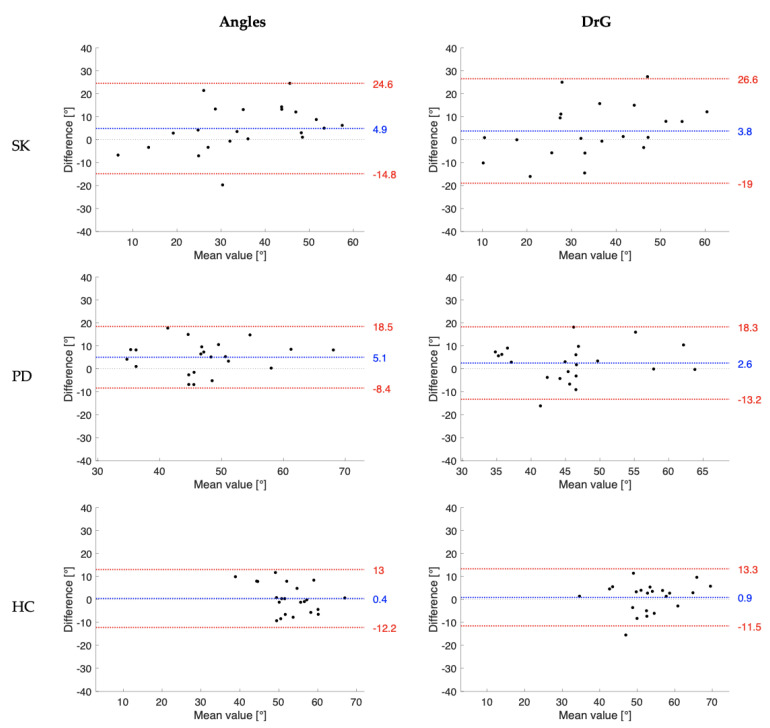
Bland-Altman plot of the knee range of motion of the three participant populations (SK: post-stroke patients, PD: patients with Parkinson’s disease, HC: healthy controls) collected by the inexperienced examiner (I1) using the Angles and DrGoniometer applications. The blue line indicates the mean differences between the mobile app and electro-goniometer measures, and the red lines are the 95% lower and upper limits of agreement.

Concurrent validity findings provided by the comparison of knee joint ROM measurements obtained by the evaluator E1 and I1 and those from the electro-goniometer are summarized in Table 4 and Figure 2 and Figure 3. For the Angles app, results showed good correlations (corr: 0.76–0.85) in the examined population samples. Comparable findings were seen for the DrGoniometer app, in which correlation coefficients ranged from 0.61 to 0.78.

Figure 2 and Figure 3 show the Bland–Altman graphic analysisfor knee joint ROM collected in the examined populations, respectively, for the experienced examiners and inexperienced examiner. Similar validity findings have been achieved using angles assessments taken by E2 and I2, as reported in Supplementary Material S1.

### 3.4. Usability

The results from the SUS showed that the mean (standard deviation) total score for the Angles app was 75 (11) points and 79 (5) for experienced and inexperienced raters, respectively. For the DrGoniometer app, the results from the SUS revealed a mean (standard deviation) total score of 79 (9) for the experienced raters and 85 (4) for the inexperienced raters.

**Figure 4:**
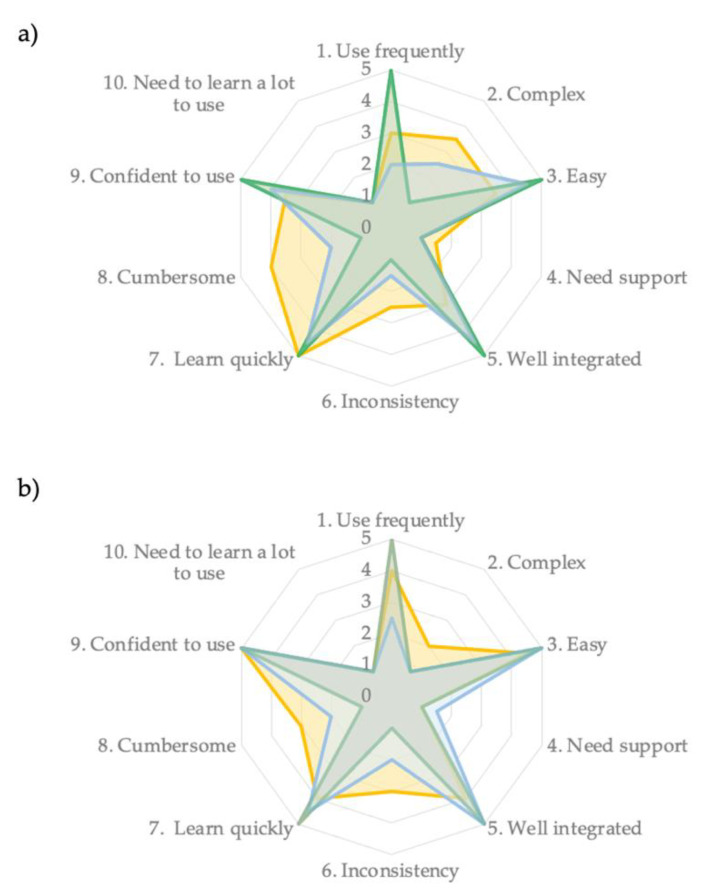
Usability findings of the Angles app (**a**) and DrGoniometer app (**b**) evaluated with the system usability scale (SUS) by experienced and inexperienced raters, respectively, in yellow and blue. The mean score collected by experienced and inexperienced raters, respectively, in yellow and blue, was reported for the ten SUS items (1: totally disagree; 5: totally agree). The green stars indicated the best possible usability results.

Figure 4 summarized the feedback about perceived system usability collected by the experienced raters and the inexperienced raters. SUS items were alternatively presented with positive (questions with odd numbers, best item score: 5) and negative (questions with even numbers, best item score: 1) statements. Overall, the majority of the users found that both apps are easy to use, and reported they did not need the assistance of a technical person to perform joint angle assessments. However, they found some inconsistencies and burdensome elements inside the apps.

## 4. Discussion

The current investigation was an attempt to examine the concurrent validity, reliability, and usability of video goniometric applications to measure the knee joint range of motion during walking. Two video goniometric applications entitled Angles and DrGoniometer were tested by independently trained clinicians (i.e., experienced examiners) and beginners who were not accustomed to these tools (i.e., inexperienced examiners). An electro-goniometer whose measurement properties were previously established was used as a comparison system [6]. Overall, the mobile apps were shown to be reasonably valid, reliable, and useful tools for assessing knee joint ROM in healthy subjects and neurological patients.

Specifically, there were good-to-excellent degrees of reliability and no systematic errors between repeated measures taken by experienced and inexperienced raters on individuals with neurological disease and healthy volunteers. These findings implied that both video goniometer apps reached clinically acceptable intra-rater reliability degrees independently of the assessors’ experience level. Indeed, intra-rater ICC showed values ranging from 0.79 to 0.95 for the experienced examiner, and analogous values (0.84–0.97) were achieved by the inexperienced examiner while using the Angles app. Acceptable intra-rater SEM and MDC values were observed in the three assessed populations for both video-based goniometer apps. Our findings generally demonstrated a slightly better intra-rater absolute reliability level for the experienced rater (SEM: 2.0–3.4°; MDC: 5.6–9.3°) than the inexperienced rater (SEM: 2.5–4.1°; MDC: 6.9–11.4°). Thus, experienced raters had a slightly lower range around the observed values within which the theoretical true score and difference lie. Our results were in line with a recent study by Cunha and colleagues [21]. They found excellent intra-rater reliability findings for the Angles app for the assessment of elbow, shoulder, hip, and knee joint angles during static and functional movements in healthy adults and children (ICC > 0.90). Our results showed lower ICC values than those reported by Cunha et al.; however, various inter-study differences in the study protocol and data analyses occur, including the studied populations (i.e., only the healthy condition), studied movements (i.e., other functional activities) and applied ICC models (i.e., the model based on a mean rating). To our knowledge, no further studies on the measurement properties of the Angles app are available, precluding possible other possible comparisons.

The DrGoniometer app revealed good-to-excellent intra-rater results when both the experienced and inexperienced examiners completed the knee joint ROM measurements. In this case, the ICC values varied from 0.85 to 0.95 for the experienced examiner, and from 0.82 to 0.94 for the inexperienced examiner. These findings were consistent with previous literature that demonstrated high intra-rater reliability when the DrGoniometer app was used in the static assessment of knee joints in healthy and pathological conditions [14,18,35]. Ferriero and colleagues [18] formerly tested the intra-rater reliability of measurements taken on a healthy volunteer at different knee joint angles, demonstrating excellent precision between repeated assessments (ICC > 0.95). Similar findings have been observed in the study of Castle et al. [35], in which experienced examiners performed good-to-excellent precise measurements during the assessment of knee angles in thirty patients who had knee replacements (ICC > 0.89).

Concerning inter-rater reliability, excellent reproducibility between examiners was found when either the Angles app or DrGoniometer app was used to assess knee joint ROM in stroke patients (ICC: 0.90–0.92). Instead, both apps revealed moderate-to-good reliable measures in patients with Parkinson’s disease and healthy volunteers (ICC: 0.69–0.84). In general, the ICC values were marginally higher between experienced raters than between inexperienced raters, whereas absolute reliability indices were smaller between experienced raters (SEM: 3.4–4.5°; MDC: 9.3–12.4°) than between inexperienced raters (SEM: 4.1–5.0°; MDC: 11.3–13.7°). In addition, systematic errors were found in the groups of patients with Parkinson’s disease and healthy volunteers between inexperienced raters. These findings might be potentially explained by the higher confidence in the identification of the anatomical landmarks of the clinicians than inexperienced raters [18]. The SEM and MDC computed for the intra-rater and inter-rater reliability were demonstrated to be clinically acceptable because they were lower than 5° and 14°, respectively [31].

Finally, significantly strong positive correlations between the measures taken from the Angles app and the electro-goniometer were found in the three examined groups (0.76–0.85). The DrGoniometer app revealed slightly lower, but still strong, correlations with the electro-goniometer measurements (0.61–0.78). Therefore, the apps could be considered a relatively valid alternative to electro-goniometers for easy and fast measurements of knee joint ROM during walking. It is important to observe from the Bland–Altman analysis that the apps consistently overestimated the knee joint angle when compared to the electro-goniometer. In addition, there were relatively wide limits of agreement for both apps, specifically for data collected on post-stroke patients due to the high inter-subject variability of angles measured in the affected leg. Conversely, the Bland–Altman plots did not reveal specific trends in the difference between methods as the mean values increased. These factors should be considered when clinicians alternatively acquire data using mobile apps and electro-goniometer. The strain gauge connecting the two end blocks of the electro-goniometer is susceptible to over-compression and torsion, which might have led to measurement errors [36]. Therefore, further investigations are needed to investigate the validity of the goniometric apps against gold-standard 3D-motion capture systems or low-cost bidimensional motion capture tools [31,37].

Several studies have shown that most of the mobile apps can provide valid reliable measurements of motion at a specific joint or region, such as the ankle, knee, wrist, or spine during passive or active movements [14,15]. The results of this study concur with recent reviews that suggested the use of smartphone-based goniometer applications in clinical practice for assessing the ROM of the knee joint [14,15]. However, although several previous studies have shown that mobile apps can provide valid reliable measurements of motion at a specific joint or region, the current study is the first to evaluate the measurement properties of video-based goniometric apps in patients with neurological diseases during walking. In addition, usability testing of goniometric apps has also received little scholarly attention and has not yet been formally established for these apps through standardized scales. Therefore, the examiners of the present study completed the SUS questionnaire at the end of the assessment period. In this study, the mean SUS scores revealed that both apps had good usability. In detail, our results indicated that experienced and inexperienced examiners felt the Angles and DrGoniometer apps were easy to understand, and they were very confident in using them; indeed, they believe that they did not need external support and additional information to learn how to use the apps. However, they were revealed to have found some inconsistencies inside the apps, making the apps slightly cumbersome. It is important to note that experienced raters would like to frequently use the DrGoniometer app more than the Angles app, probably thanks to its user interface design and functioning specifically developed for clinical purposes.

The present study is associated with some limitations that should be considered. The study included two examiners per group for testing the inter-rater reliability and only one examiner per group for intra-rater reliability evaluation. Future studies should increase the number of examiners to statistically compare reliability between experienced and inexperienced examiners.

Each participant was evaluated on a single occasion, and if this test was repeated on a second day, it could provide additional information about the test–retest reliability of the measurements. In future studies, test–retest reliability assessment of mobile goniometer apps during walking is suggested. In the present study, smartphone videos were captured by trained examiners, and not by the patient’s caregiver in a remote setting. Therefore, our results should also be investigated in a completely uncontrolled scenario at patients’ home to increase the external validity and generalizability of our results. Our study was limited to the assessment of two specific but relevant knee joint angles (i.e., maximum flexion and maximum extension) during a gait cycle. For each angle, the examiner had to import the video recording, select a video frame and place three movable markers on the smartphone screen. The higher the number of joint angles the examiner wants to assess in a gait cycle, the more time-consuming the video-based goniometer apps will be. For time and resource reasons, the current study was focused on and limited to the knee joint only. The decision to choose knee joint ROM was made considering the typical gait impairments of the studied neurological populations (i.e., stiff knee gait and knee bending due to postural deformities [38,39,40]). Further studies are encouraged to examine the measurement properties of ROM measures collected in other joints.

Another limitation was the use of an electro-goniometer as a comparison tool. Although this instrument is a relatively inexpensive solution for clinical settings, it could be subjected to measurement errors itself. Further studies are needed to validate the present two apps with a gold standard marker-based motion capture system.

Nevertheless, one strength of our study was that the entire measurement procedure was performed in a clinical condition. Therefore, we did not use a common set of frames taken by the video for each examiner, as were used in the previous studies; our raters had to independently select the frame from the video and consequently assess the knee joint ROM in it. The results of the present study consequently included variability in frame selection and then in angle estimates. Other strengths of this study involved the comparison of multiple mobile apps, blinding of the assessors’ measurements, assessments performed by raters with different experience levels, and the inclusion of participants with different pathological conditions and different joint knee limitations.

In conclusion, the Angles and DrGoniometer apps represent a new measurement approach for measuring knee joint range of motion during walking, but further research should be encouraged to analyze measurement properties, using these mobile applications on (i) different functional movements, (ii) different joints, (iii) different pathological conditions, and (iv) videos from remote settings, taken by caregivers or patients themselves, to fill the gap in knowledge in the literature and increase the generalizability of our findings.

## Figures and Tables

**Table 1:** The main characteristics of the participants.

	SK	PD	HC
Sample size	22	22	22
Age [years]	61 (13)	72 (8)	45 (5)
Sex [male/female]	17/5	18/4	9/13
Time since diagnosis [years]	1 (2)	5 (4)	-
Ictus etiology [isch/hem]	18/4	-	-
Hemiparesis [R/L]	14/8	-	-
mRS scale [0–5]	2 (1)		
mH&Y scale [1–5]	-	2 (1)	-

SK: stroke; PD: Parkinson’s disease; HC: healthy control; Isch: ischemic; Hem: hemorrhagic; R: right; L: left; mRS: modified Rankin scale; mH&Y: modified and Yahr scale. Continuous data are presented using mean (standard deviation), and binary data using absolute numbers.

**Table 2:** Mean values and intra-rater reliability of range of motion measurements acquired by the Angles and DrGoniometer applications by the experienced and the inexperienced raters in the three studied populations.

		ROM [°]				*p*-Value (T1 vs. T2)		ICC [95% CI]		SEM [°]		MDC [°]	
App	Population	E1		I1		E1	I1	E1	I1	E1	I1	E1	I1
		T1	T2	T1	T2								
Angles	SK	37.9 (14.5)	39.4 (12.7)	35.4 (15.6)	35.3 (11.7)	0.11	0.85	0.95 [0.87; 0.98]	0.97 [0.92; 0.99]	3.2	2.5	8.8	6.9
	PD	48.4 (7.5)	49.3 (9.1)	46.5 (9.7)	47.9 (8.5)	0.39	0.18	0.87 [0.71; 0.94]	0.88 [0.73; 0.95]	3.1	3.2	8.5	9.0
	HC	54.8 (5.3)	53.8 (6.5)	54.2 (9.4)	54.3 (6.9)	0.24	0.90	0.79 [0.56: 0.91]	0.84 [0.65; 0.93]	2.7	3.3	7.5	9.2
DrG	SK	37.8 (16.1)	37.0 (13.7)	36.7 (17.1)	36.5 (16.9)	0.42	0.93	0.95 [0.88; 0.98]	0.94 [0.86; 0.98]	3.4	4.1	9.3	11.4
	PD	49.8 (9.2)	48.0 (7.9)	47.2 (9.3)	47.4 (8.3)	0.08	0.92	0.85 [0.67; 0.94]	0.82 [0.62; 0.92]	3.3	3.7	9.2	10.3
	HC	53.1 (5.8)	52.4 (7.3)	53.6 (9.2)	54.4 (6.8)	0.23	0.48	0.91 [0.79; 0.96]	0.82 [0.61; 0.92]	2.0	3.5	5.6	9.6

Angles: Angles - Video Goniometer application; DrG: Dr Goniometer application; SK: stroke; PD: Parkinson’s disease; HC: healthy control; ROM: Range Of Motion; E1: Experienced examiner number 1; I1: Inexperienced examiner number 1; ICC: intraclass correlation coefficient; CI: confidence interval; T1: visit 1; T2: visit 2; SEM: standard error of measurement; MDC: minimal detectable change. Continuous ROM data are presented as mean (standard deviation).

**Table 3:** Inter-rater reliability of ROM measures from the Angles and DrGoniometer applications.

App	Population	Rater	ROM - Rater 1 [°]	ROM - Rater 2 [°]	*p*-Value	ICC [95% CI]	SEM [°]	MDC [°]
Angles	SK	E	37.9 (14.5)	40.2 (13.3)	0.09	0.90 [0.76; 0.96]	4.5	12.4
		I	35.4 (15.6)	33.8 (18.5)	0.28	0.92 [0.81; 0.96]	5.0	13.7
	PD	E	48.4 (7.5)	50.7 (10.9)	0.06	0.80 [0.58; 0.92]	4.1	11.5
		I	46.5 (9.7)	46.6 (9.1)	0.95	0.75 [0.48; 0.89]	4.7	13.1
	HC	E	54.8 (5.3)	56.2 (7.1)	0.17	0.71 [0.44; 0.87]	3.4	9.3
		I	54.2 (9.4)	54.1 (6.6)	0.95	0.75 [0.49; 0.89]	4.1	11.3
DrG	SK	E	37.8 (16.1)	37.4 (13.6)	0.81	0.92 [0.82; 0.97]	4.1	11.5
		I	36.7 (17.1)	36.8 (16.4)	0.89	0.92 [0.82; 0.97]	4.7	13.0
	PD	E	49.8 (9.2)	48.6 (10.0)	0.34	0.84 [0.65; 0.93]	3.9	10.8
		I	47.2 (9.3)	51.3 (8.4)	0.005 *	0.71 [0.30; 0.88]	4.8	13.4
	HC	E	53.1 (5.8)	55.4 (7.7)	0.05	0.70 [0.40; 0.87]	3.7	10.3
		I	53.6 (9.2)	57.4 (8.4)	0.01 *	0.69 [0.32; 0.86]	4.9	13.6

Angles: Angles - Video Goniometer application; DrG: Dr Goniometer application; SK: stroke; PD: Parkinson’s disease; HC: healthy control; E: experienced examiner; I: inexperienced examiner; ICC: intraclass correlation coefficient; CI: confidence interval; SEM: standard error of measurement; MDC: minimal detectable change; ROM: Range Of Motion. * *p*-value < 0.05. Continuous ROM data are presented as mean (standard deviation).

**Table 4:** Concurrent validity of the range of motion measurements acquired by the Angles and DrGoniometer applications in the three studied populations, and agreement between expert and non-expert assessments (E1, I1) with respect to the electro-goniometer.

Population	Rater	ROM - Angles App [°]	ROM - DrG App [°]	Electro- Goniometer [°]	Angles App vs. Electro-Goniometer		DrG App vs. Electro-Goniometer	
					Mean Bias [°] [95%LoA]	Corr	Mean Bias [°] [95%LoA]	Corr
SK	E1	37.9 (14.5)	37.8 (16.1)	32.7 (12.7)	4.1 [−13.6; 21.8]	0.78	2.5 [−13.9; 18.9]	0.78
	I1	35.4 (15.6)	36.7 (17.1)		4.9 [−14.8; 24.6]	0.85	3.8 [−19.0; 26.6]	0.73
PD	E1	48.4 (7.5)	49.8 (9.2)	44.7 (8.7)	3.7 [−7.6; 15.0]	0.76	1.8 [−10.6; 14.3]	0.71
	I1	46.5 (9.7)	47.2 (9.3)		5.1 [−8.4; 18.5]	0.77	2.6 [−13.2; 18.3]	0.61
HC	E1	54.8 (5.3)	53.1 (5.8)	52.7 (8.0)	2.1 [−6.8; 11.0]	0.85	1.5 [−9.2; 12.1]	0.67
	I1	54.2 (9.4)	53.6 (9.2)		0.4 [−12.2; 13.0]	0.82	0.9 [−11.5; 13.3]	0.74

Angles app: Angles - Video Goniometer application; DrG app: Dr Goniometer application; SK: stroke; PD: Parkinson’s disease; HC: healthy control; E1: experienced examiner number 1; I1: inexperienced examiner number 1; LoA: limits of agreement; Corr: correlation coefficient; ROM: Range Of Motion. Continuous data are presented as mean (standard deviation).

## Data Availability

Data contained in this study are available on reasonable request from corresponding author.

## Supplementary Materials

The following supporting information can be downloaded at: https://www.mdpi.com/article/10.3390/s23042232/s1, Figure S1: Bland-Altman plot of the knee range of motion of the three participants’ populations (SK: post-stroke patients, PD: patients with Parkinson’s disease, HC: healthy controls) collected by the experienced examiner (i.e. E2) using the Angles and the DrGoniometer applications; Figure S2: Bland-Altman plot of the knee range of motion of the three participants’ populations (SK: post-stroke patients, PD: patients with Parkinson’s disease, HC: healthy controls) collected by the inexperienced examiner (i.e. I2) using the Angles and the DrGoniometer applications; Table S1: Concurrent validity of the range of motion measurements acquired by Angles and DrGoniometer apps in the three studied populations and agreement between experienced and inexperienced assessments (E2, I2) with respect to the electro-goniometer.
