# Supplementary material for: Video-based Goniometer Applications for Measuring Knee Joint Angles during Walking in Neurological Patients: A Validity, Reliability and Usability Study
Source: Sensors (Basel). 2023 Feb 16;23(4):2232. doi: 10.3390/s23042232 (PMC9960424; doi:10.3390/s23042232)
Supplement: Supplementary file 1 [file sensors-23-02232-s001.zip › sensors-2101002-supplementary.pdf]

Table S1. Concurrent validity of the range of motion measurements acquired by Angles and DrGoniometer apps in the three studied populations and agreement between experienced and unexperienced assessments (E2, I2) with respect to the electro-goniometer.

Population	Rater	ROM - Angles App [°]	ROM - DrG App [°]	Electro-goniometer [°]	Angles app vs Electro-goniometer		DrG app vs Electro-goniometer	
					Mean Bias [°] [95%LoA]	Corr	Mean Bias [°] [95%LoA]	Corr
SK	E2	40.2 (13.3)	37.4 (13.6)	32.7 (12.7)	6.5 [-8.0; 20.9]	0.84	4.6 [-13.6; 22.7]	0.75
	I2	33.8 (18.5)	36.8 (16.4)		0.9 [-23.1; 24.9]	0.75	4.0 [-18.9; 26.8]	0.71
PD	E2	50.7 (10.9)	48.6 (10.0)	44.7 (8.7)	6.1 [-6.4; 18.5]	0.81	3.9 [-12.1; 20.0]	0.63
	I2	46.6 (9.1)	51.3 (8.4)		1.9 [-10.5; 14.4]	0.74	6.6 [-6.9; 20.1]	0.68
HC	E2	56.2 (7.1)	55.4 (7.7)	52.7 (8.0)	3.5 [-7.5; 14.5]	0.73	2.7 [-9.8; 15.1]	0.68
	I2	54.1 (6.6)	57.4 (8.4)		1.4 [-12.2; 14.9]	0.57	4.6 [-6.5; 15.8]	0.76

Angles app: Angles – video goniometer application; DrG app: Dr Goniometer application; SK: stroke; PD: Parkinson's Disease; HC: Healthy Control; E2: Experienced examiner number 2; I2: Inexperienced examiner number 2; LoA: Limits of Agreeement; Corr: Correlation Coefficient; ROM: Range Of Motion. Continuous data are presented as mean (standard deviation).

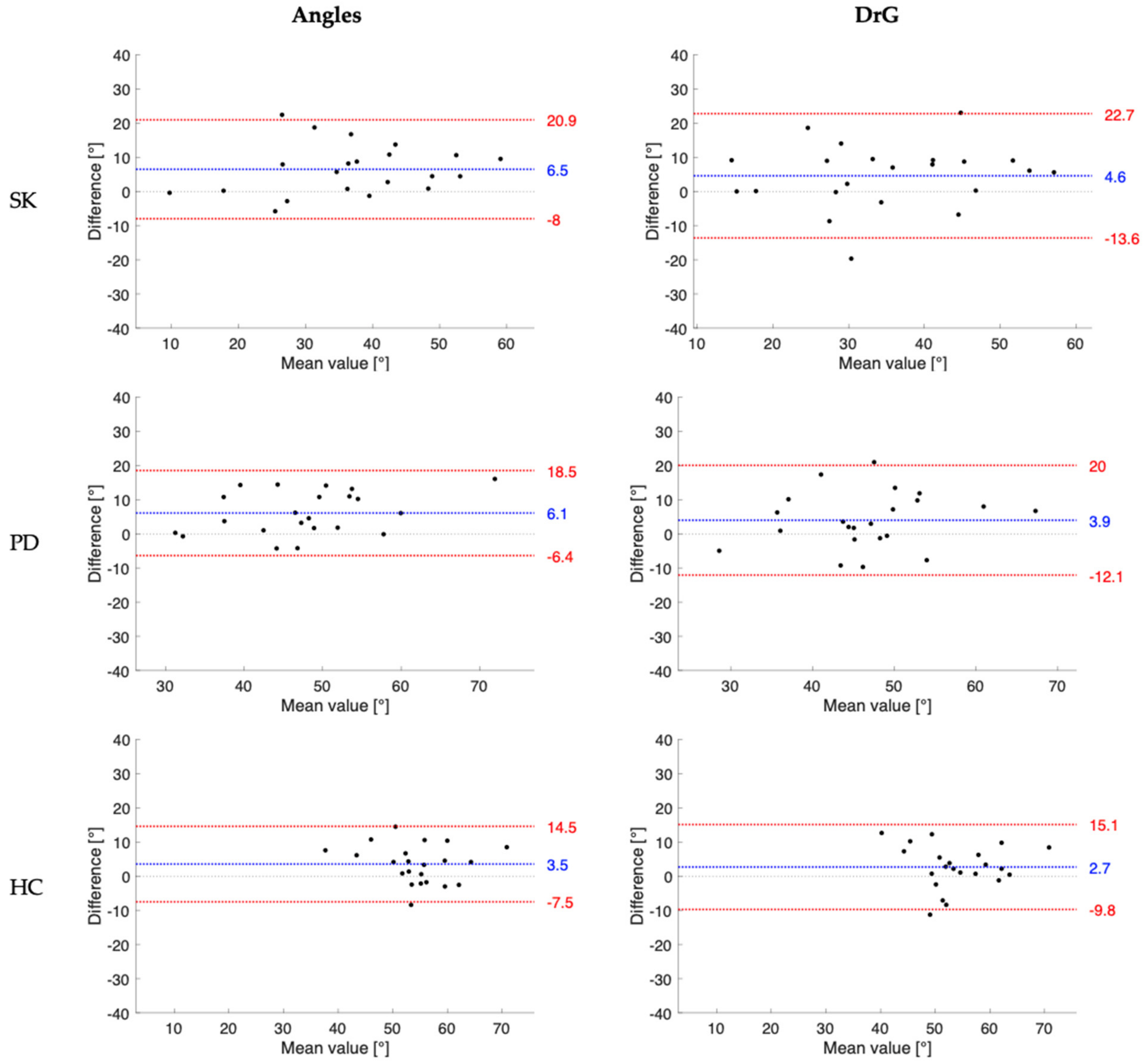

Figure S1. Bland-Altman plot of the knee range of motion of the three participants' populations (SK: post-stroke patients, PD: patients with Parkinson's disease, HC: healthy controls) collected by the experienced examiner (i.e. E2) using the Angles and the DrGoniometer applications. The blue line indicates the mean differences between a mobile app and electrogoniometer measures, the red lines are the 95% lower and upper limits of agreement.

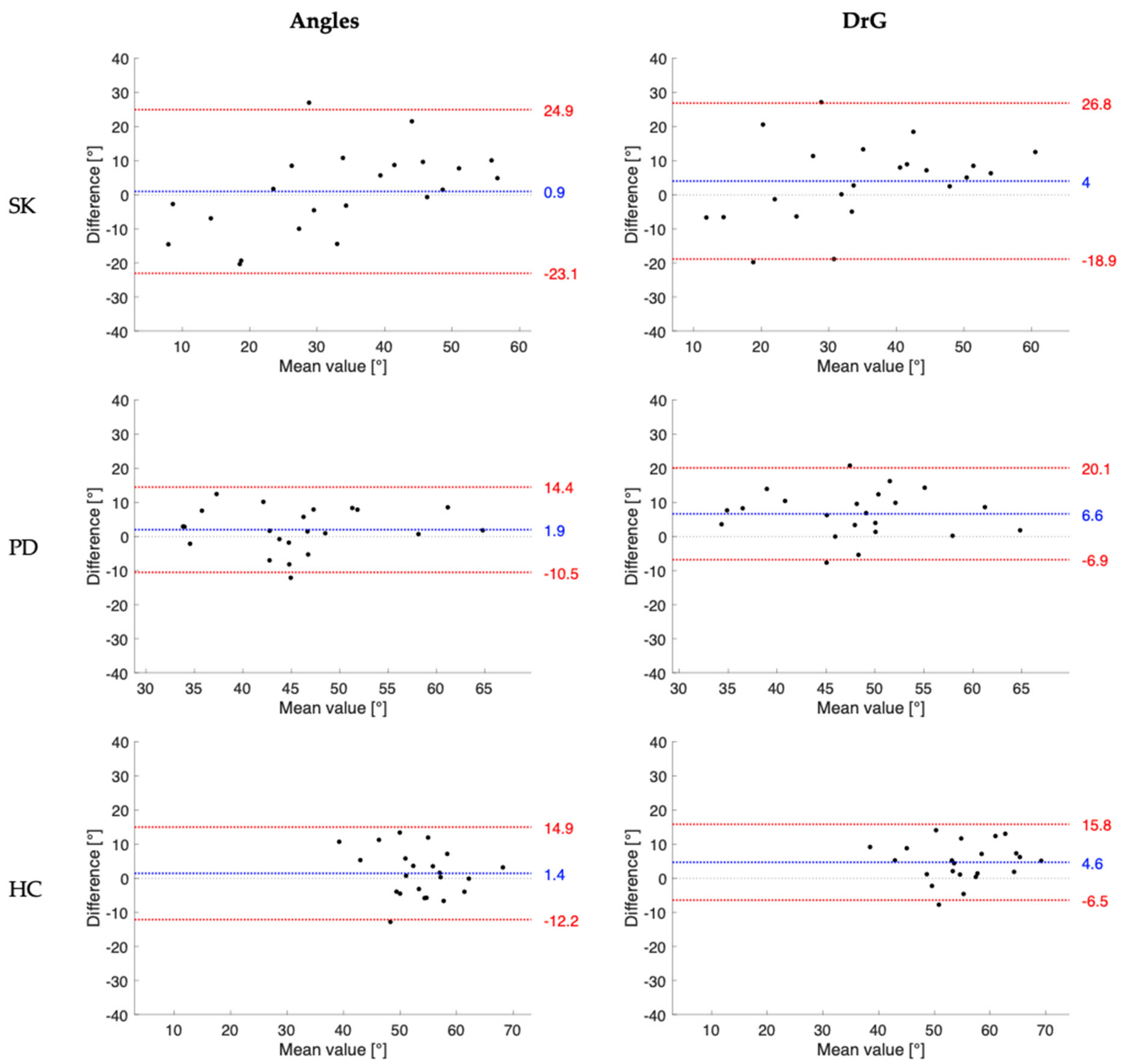

Figure S2. Bland-Altman plot of the knee range of motion of the three participants' populations (SK: post-stroke patients, PD: patients with Parkinson's disease, HC: healthy controls) collected by the inexperienced examiner (i.e I2) using the Angles and the DrGoniometer. The blue line indicates the mean differences between a mobile app and electro-goniometer measures, the red lines are the 95% lower and upper limits of agreement.
